# Modeling the Electrochemical Synthesis of Zinc Oxide Nanoparticles Using Artificial Neural Networks

**DOI:** 10.3390/ma18174187

**Published:** 2025-09-06

**Authors:** Sławomir Francik, Michał Hajos, Beata Brzychczyk, Jakub Styks, Renata Francik, Zbigniew Ślipek

**Affiliations:** 1 Department of Mechanical Engineering and Agrophysics, Faculty of Production Engineering and Energetics, University of Agriculture in Krakow, Al. Mickiewicza 21, 31-120 Krakow, Poland; beata.brzychczyk@urk.edu.pl (B.B.); jakub.styks@urk.edu.pl (J.S.); 2 Faculty of Medicine and Health Sciences, University of Applied Sciences in Nowy Sacz, Kosciuszki 2G, 33-300 Nowy Sacz, Poland; rfrancik@ans-ns.edu.pl; 3 Faculty of Engineering Sciences, University of Applied Sciences in Nowy Sacz, Zamenhofa 1a, 33-300 Nowy Sacz, Poland; zslipek@ans-ns.edu.pl

**Keywords:** Artificial Intelligence, ANN model, ZnO nanoparticle, electrochemical synthesis of nanoparticles, nanoparticles size distribution

## Abstract

A neural model was developed to predict the distribution of ZnO nanoparticles obtained by electrochemical synthesis. It is a three-layer multilayer perceptron (MLP) artificial neural network (ANN) with five neurons in the input layer, eight neurons in the hidden layer, and one neuron in the output layer. This network has a hyperbolic tangent activation function for the neurons in the hidden layer and an exponential activation function for the neuron in the output layer. The input (independent) variables are particle size (nm), solvent type, and temperature (°C), and the output (dependent) variable is fraction share (%). The best neural model (ann08) has a root mean square error (RMSE) 0.84% for the training subset, 0.98% for the testing subset, and 1.27% for the validation subset. The RMSE values are therefore small, which enables practical use of the ANN model.

## 1. Introduction

Nanotechnology is gaining increasing importance in a wide variety of application areas. Nanoparticles are successfully used in various fields, such as electronics, power engineering, environmental engineering, medicine, the pharmaceutical industry, cosmetics, and textiles [1,2,3]. The physical, chemical, and biological properties of metal oxide nanoparticles are of particular interest, including zinc oxide nanoparticles.

Zinc oxide, also found in a nanometric form, has a number of distinctive properties. It is an n−type semiconductor, which, in combination with p-type semiconductors, forms the basis for the construction of transistors, LEDs, and photovoltaic cells [4,5,6]. The electrical properties of zinc oxide are also used in the construction of gas detectors [7].

The most common applications of zinc oxide utilize its optical properties, namely its ability to absorb and reflect UV radiation. Therefore, ZnO is often an ingredient in sunscreen creams [8,9,10] and in paints and varnishes [11,12], both as a white pigment and as an ingredient that provides protection against UV rays and increases durability.

The antibacterial properties of zinc oxide are also significant. Combined with its drying and astringent properties, zinc oxide can be used in dressings and ointments to promote wound healing [10,13,14,15,16,17,18,19] as well as in antibacterial and antifungal protective fabrics [20,21,22].

Its antiseptic properties also allow zinc oxide to be used in the manufacture of food packaging [15,23,24], as these nanoparticles have been recognized as safe by the U.S. Food and Drug Administration [2,3]. 

The process of electrochemical synthesis of zinc oxide nanoparticles using anodic dissolution of metallic zinc is a promising method of producing zinc oxide nanoparticles. It can successfully compete with or be an alternative to currently developed and commonly used methods of producing metal and/or metal oxide nanoparticles [25,26,27,28,29].

The electrochemical synthesis method for producing ZnO nanoparticles is widely used due to its simplicity, low operating temperature, low energy consumption, and the high purity of the synthesized product [30].

Electrochemical synthesis takes place in electrolytes, which are alcoholic solutions of salts with the addition of water. Under the influence of an external current source, the zinc electrode is immersed in the electrolyte, which is the anode of the cell, and is dissolved. Zinc ions detach from the anode surface and pass into the solution. As a result of electrode reactions and subsequent reactions, a suspension of zinc oxide nanoparticles in an alcohol solution is formed. During the synthesis, the reaction medium is purged with an inert gas (argon) in order to deoxygenate the solution and mix it. A challenge in using this method of obtaining zinc oxide nanoparticles (but also other metals and oxides) may be the contamination of the final product with chloride ions originating from salts in the electrolyte, adsorbed on the surface of the nanoparticles. This problem can be easily eliminated by rinsing the nanoparticle suspension with pure alcohol, which is the basis of the solution used for synthesis [31]. 

The described method of zinc oxide nanoparticle electrochemical synthesis is environmentally friendly and does not generate hazardous waste. Apart from a direct current, which is necessary for electrode reactions, it does not require significant energy input. Even ensuring a process temperature higher than the ambient temperature, using a water bath, does not significantly increase the energy consumption of the process. In order to maintain a stable electrolyte composition, the synthesis of zinc oxide nanoparticles was carried out below the boiling point of the first alcohol in the series—methanol, at a maximum temperature of 50 °C [30,32]. 

In previous works on the described method of zinc oxide nanoparticle synthesis, it was shown that the process of their electrochemical production depends on many factors. The type of alcohol, the type and concentration of the anion derived from the salt, the quantitative addition of water, the temperature of the process [33], and the current values [34,35] influence the structure, morphology, purity, and properties of the obtained product. Depending on the values of the parameters used to synthesize nanoparticles, nanoparticles of different sizes/size distributions can be obtained. The main role here is played by the type of alcohol solvent [25,26,27], but also by the process temperature [33].

The analysis of the size distributions of zinc oxide nanoparticles formed in solutions with different solvents and at different temperatures can lead to the application of a set of synthesis process parameters that will result in nanoparticles of the desired size, i.e., enable process control. Similar observations regarding the production of nanoparticles and process control through parameter adjustment are presented in the work by Balanov et al. [36]. Despite using a different method (hydrothermal method), the researchers encountered challenges similar to those associated with the electrochemical method, concerning particle agglomeration and changes in composition. However, the authors emphasize the advantage of the method, which is the possibility of controlling the process by means of the pH of the solution and the process temperature.

The traditional experimental research approach, although necessary, may prove troublesome. Considering the number of possible parameters of electrochemical synthesis of zinc oxide nanoparticles, when changing the value of just one of them, it is necessary to prepare a new electrolyte solution and conduct a new synthesis. Only after the completion of the nanoparticle production process is it possible to determine the effect of the selected, changed parameter on the size distribution or form of the formed particles.

Scientists are searching for tools that will allow them to model the electrochemical synthesis of ZnO nanoparticles. Such models will allow them to optimize the process in terms of productivity and consumption [30].

One of the most effective and widely used tools for modeling various processes is artificial neural networks (ANNs). ANNs have been successfully used in many different research areas: engineering, energy, biochemistry, bioinformatics, medicine, agricultural engineering, economics, and meteorology [37,38,39,40,41,42,43,44,45,46,47,48]. 

ANNs are also being successfully used in the field of nanotechnology.

Huanbutta et al. analyzed the applications of artificial intelligence methods in the pharmaceutical industry, particularly the application of AI in the design, characterization, and manufacturing of drug delivery nanosystems [49].

In their review article, Jeznach et al. described the applications of artificial intelligence tools in tissue engineering, including the optimization of bioprinting; the prediction of material–cell interactions; the optimization of drug delivery systems; the prediction of the toxicity of nanoparticles; the design of innovative drug delivery systems, including intelligent drug delivery; and the use of implantable microchips and nanomaterials as drug reservoirs of various geometries [50].

Basu et al. reviewed the current state of research on the use of artificial intelligence methods to predict the thermophysical properties of nanofluids used in heat transport [51].

Below are examples of the use of artificial neural networks in nanotechnology:

- Ma et al. developed two ANNs for predicting the thermophysical parameters (thermal conductivity and viscosity) of hybrid nanofluids (Al_2_O_3_ nanoparticles, CuO nanoparticles, ethylene glycol, water) [52].
- Zhao and Li developed a radial basis function neural network to predict thermal conductivity and viscosity for alumina–water nanofluids [53].
- Zhu et al. developed an ANN to predict the thermal conductivity of ethylene glycol (EG) and alumina (Al_2_O_3_) nanofluids [54].
- Hou et al. developed a backpropagation artificial neural network (BP-ANN) to model the removal efficiency of ethyl violet in wastewater using reduced graphene oxide modified with manganese-doped iron nanoparticles [55].
- Ruan et al. developed ANNs to model the removal of crystal violet from aqueous solutions using bimetallic Fe/Ni nanoparticles supported by reduced graphene oxide [56].
- Khatamian et al. developed a three-layer backpropagation neural network to model the performance of the catalytic degradation process of 4-nitrophenol using ZnO nanoparticles supported on zeolites (ZnO-HZSM-5) [57].
- Melaibari et al. developed an ANN to predict the viscosity of an antifreeze hybrid nanofluid (water–ethylene glycol) containing graphene oxide and copper oxide [58].
- Boateng et al. developed a residual neural network (ResNet) to predict the size of standard nanoparticles and extracellular vesicles [59].
- Smeraldo et al. used ANN to model the effect of microfluidic parameters on nanoparticle size [60].
- Zhou et al. used a unidirectional ANN to predict the changes in crystal size, ultimate stress, and antibacterial activity of zinc oxide nanoparticles [61].
- Vaferi et al. developed multilayer perceptron neural networks to predict the size of alumina agglomerations in water-based nanofluids [62].
- Ragone et al. used convolutional neural networks (CNNs) to detect the atomic column height of gold nanoparticles from high-resolution transmission electron microscopy images [63].
- Frages et al. used ANNs for shape classification of nanoparticles described as 3D models [64].
- Zelenka et al. developed a deep ANN for measuring and classifying bi-metallic nanoparticles (Au-Co and Au-Fe) [65].
- Ijaz et al. developed an ANN to model the synthesis of optimized ZnO nanoflowers [66].
- Liu et al. used ANN to model the effect of iron and nickel nanoparticle additions on the biohydrogen production process (yield and hydrogen evolution rate) [67].
- Muneer et al. developed an ANN to simulate the zeta potential of silica nanofluids [68].

The use of artificial neural networks (ANN) as a simulation and prediction tool becomes absolutely justified in the case of modeling the electrochemical synthesis process of zinc oxide nanoparticles. Thanks to the ability to learn from available data and model non-linear dependencies, they can effectively support the synthesis process, not only from the point of view of optimizing process parameters. The use of neural networks may also allow for controlling the synthesis process in order to obtain a product with, for example, the desired size distribution.

Based on the values of the input parameters of the process, the operation of artificial neural networks will allow us to predict the results of the experiment without the need to conduct real experiments every time.

ANN-based simulations can also help determine the parameters that have the greatest impact on the process, from the point of view of the quality and size of the resulting ZnO particles. It is also possible to create adaptive models that learn as more experimental data are provided, which increases their accuracy and practical application.

The aim of this work was to develop a model using artificial neural networks that will enable the prediction of ZnO nanoparticle distribution obtained in the electrochemical synthesis process.

## 2. Materials and Methods

The model using artificial neural networks was created in accordance with the methodology developed in previous studies and proven [39,40], which includes several steps:

- Semantic model formulation;
- Selecting neural network types and carrying out the process of learning;
- Choosing and assessing the best neural models.

The data used to create the neural models were the results of experimental measurements [33].

### 2.1. Semantic Model Formulation

For the purposes of our work, the following independent variables were assumed as SNN inputs: ZnO particle size (nm), solvent type, and the temperature at which the synthesis was carried out (°C). Particle size and temperature were quantitative variables. Solvent type was a qualitative variable that used three values: methanol, ethanol, and propanol.

The output of the artificial neural network (dependent variable) was the fraction share, expressed as a percentage, of particles of a given size obtained for a given value of temperature and solvent type.

The adopted semantic model took the form:

*FS* = f (*PS*, *ST*, *T*) (1)

where *FS*—Fraction Share (%); 

*PS*—Particle Size (nm);

*ST*—Solvent Type (-);

*T*—temperature (°C).

### 2.2. Selecting Neural Network’s Type and Carrying out the Process of Learning

A significant problem in creating neural models is the choice of the type of ANN. In the Statistica Neural Networks program, multilayer perceptron (MLP) networks and radial basis function networks can be used to create neural models. Our preliminary (unpublished) studies have shown that better results (lower error values) are obtained using MLP networks, which is why this type of network was chosen to develop the neural model.

The next problem was the selection of the activation function in the hidden layer and the output layer. Three combinations of activation functions were compared:

- Hyperbolic tangent function in the hiding layer and exponential function in the output layer;
- Hyperbolic tangent function in the hiding layer and logistic function in the output layer;
- Hyperbolic tangent function in the hiding layer and hyperbolic tangent function in the output layer.

For these three variants, the root mean square error (RMSE) values were calculated for the best neural models. For the hyperbolic tangent function and exponential function variants, the RMSE was 0.84% for the training subset, 0.98% for the testing subset, and 1.27% for the validation subset, respectively. For the hyperbolic tangent function and logistic function variants, the RMSE was 1.19% for the training subset, 1.61% for the testing subset, and 1.88% for the validation subset, respectively. For the hyperbolic tangent function and hyperbolic tangent function variants, the RMSE was 1.64% for the training subset, 1.97% for the testing subset, and 1.96% for the validation subset, respectively.

The results of the comparison of these variants showed that the most accurate models can be obtained for hyperbolic tangent function in the hiding layer and exponential function in the output layer. Therefore, these activation functions were selected to further create the resulting neural models.

The formulas of the individual activation functions are given in Table 1.

**Figure 1:**
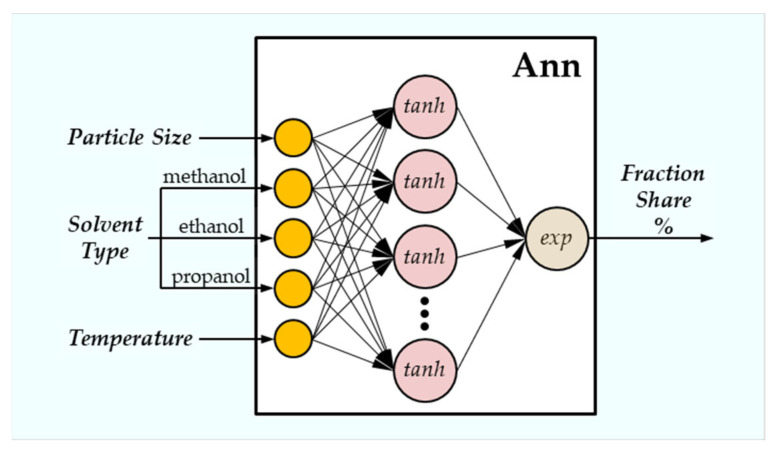
The diagram of the artificial neural network architecture.

The architectures of artificial neural networks for which the neural models were developed had five input neurons, between four and ten neurons in the hidden layer, and one output neuron. The diagram of the network architecture is presented in Figure 1.

To create the ANN models, 384 cases (data−experimental measurement results) were used, which were randomly divided into three subsets: learning, testing, and validation at proportions of 75%, 15%, and 15%.

While undertaking the ANN learning process using the “Automatic Designer” function, the process was repeated many times for different numbers of neurons in the hidden layer (100 repetitions). The 10 most accurate neural models were retained for further analysis.

### 2.3. Choosing and Assessing the Best Neural Models

The selection of the best artificial neural network was made based on the root mean square error (RMSE) values for the test subset, calculated using the formula [39,40,48,53,54,60,62,68]:

(2) $$RMSE=\sqrt{\frac{1}{n}\sum_{i=1}^{n}{\left({FS}_{ME,i}-{FS}_{ANN,i}\right)}^{2}}$$

where

*RMSE*—root mean-square error (%),

*FS_ME,i_*—measured value of output (%),

*FS_ANN,i_*—calculated by ANN value of output (%),

*n*—number of observations (-).

### 2.4. Sensitivity Analysis

A sensitivity analysis was performed to assess the importance of individual input variables [39,40]. This analysis allows us to determine the importance of independent variables for model accuracy. The error ratio was used. The higher the error ratio, the greater the importance of the variable for model accuracy.

## 3. Results

**Figure 2:**
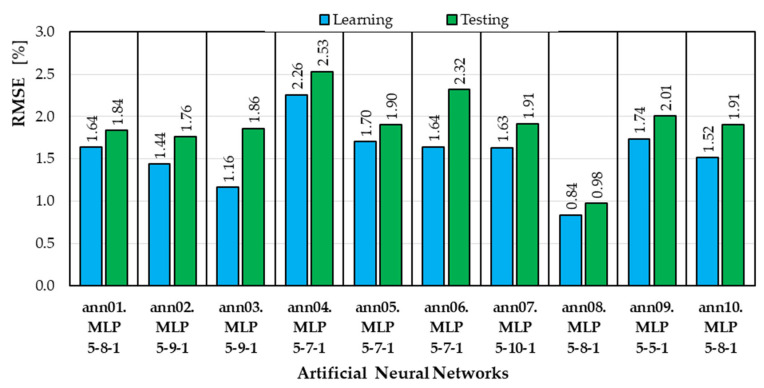
RMSE values for the learning and testing subsets.

The RMSE values for the learning and testing subsets for the top 10 retained neural models are shown in Figure 2.

The best model turned out to be the ann08 neural network (MLP 5-8-1) with eight neurons in the hidden layer, which for the testing subset obtained an RMSE of 0.98%.

**Figure 3:**
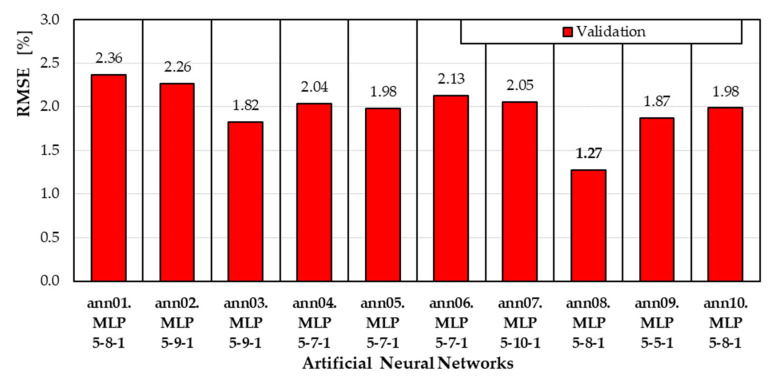
The RMSE values for the validation subset.

The RMSE values for the validation subset are shown in Figure 3.

**Figure 4:**
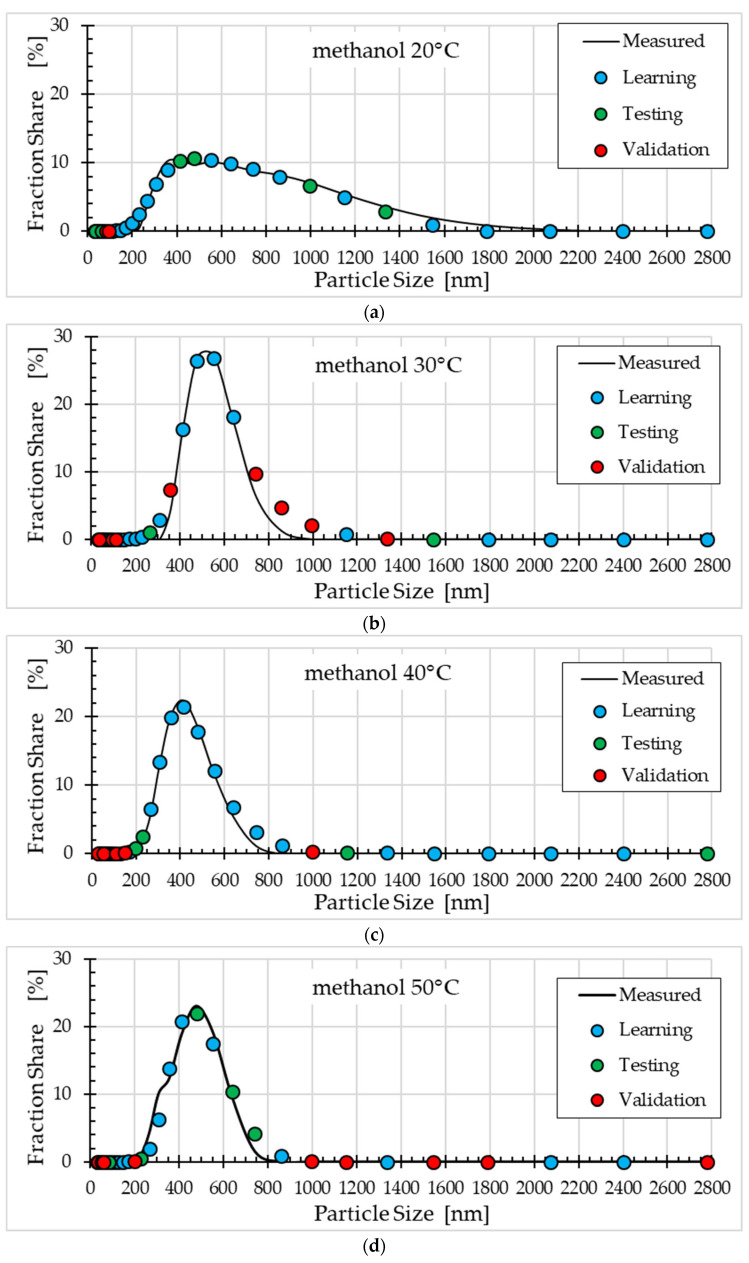
Comparison of fraction share values measured and obtained using the ann08 (MLP 5−8−1) neural model for methanol: (**a**) synthesis at 20 °C, (**b**) synthesis at 30 °C, (**c**) synthesis at 40 °C and (**d**) synthesis at 50 °C.

**Figure 5:**
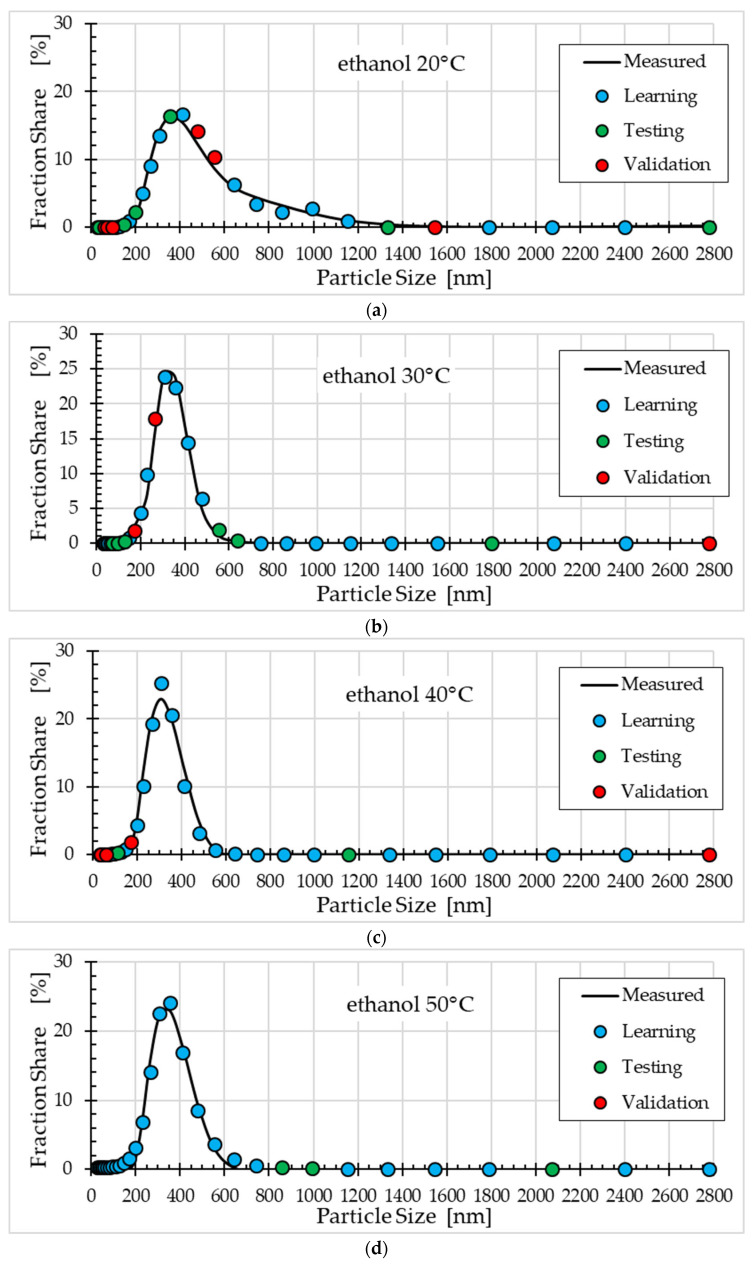
Comparison of fraction share values measured and obtained using the ann08 (MLP 5−8−1) neural model for ethanol: (**a**) synthesis at 20 °C, (**b**) synthesis at 30 °C, (**c**) synthesis at 40 °C and (**d**) synthesis at 50 °C.

**Figure 6:**
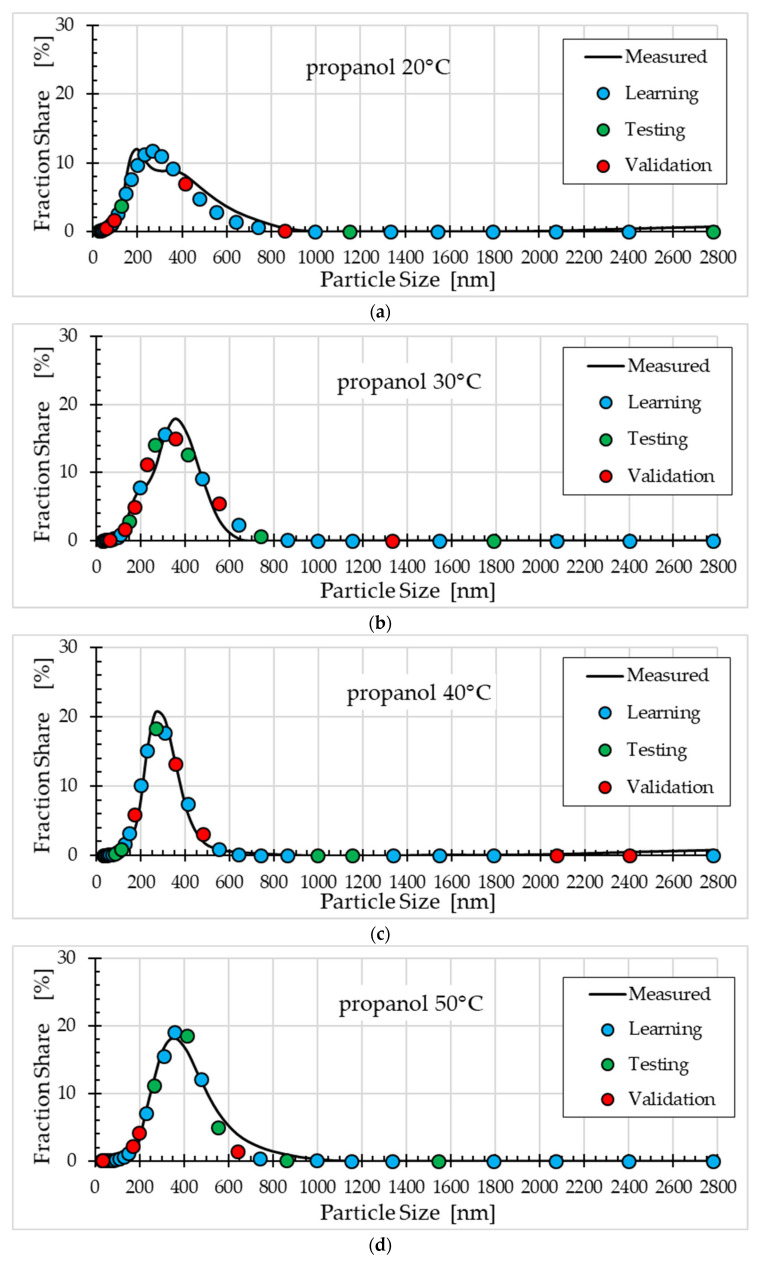
Comparison of fraction share values measured and obtained using the ann08 (MLP 5−8−1) neural model for propanol: (**a**) synthesis at 20 °C, (**b**) synthesis at 30 °C, (**c**) synthesis at 40 °C and (**d**) synthesis at 50 °C.

Figure 4, Figure 5 and Figure 6 compare the fraction share values measured and obtained using the selected ann08 (MLP 5-8-1) neural model for different solvents.

**Figure 7:**
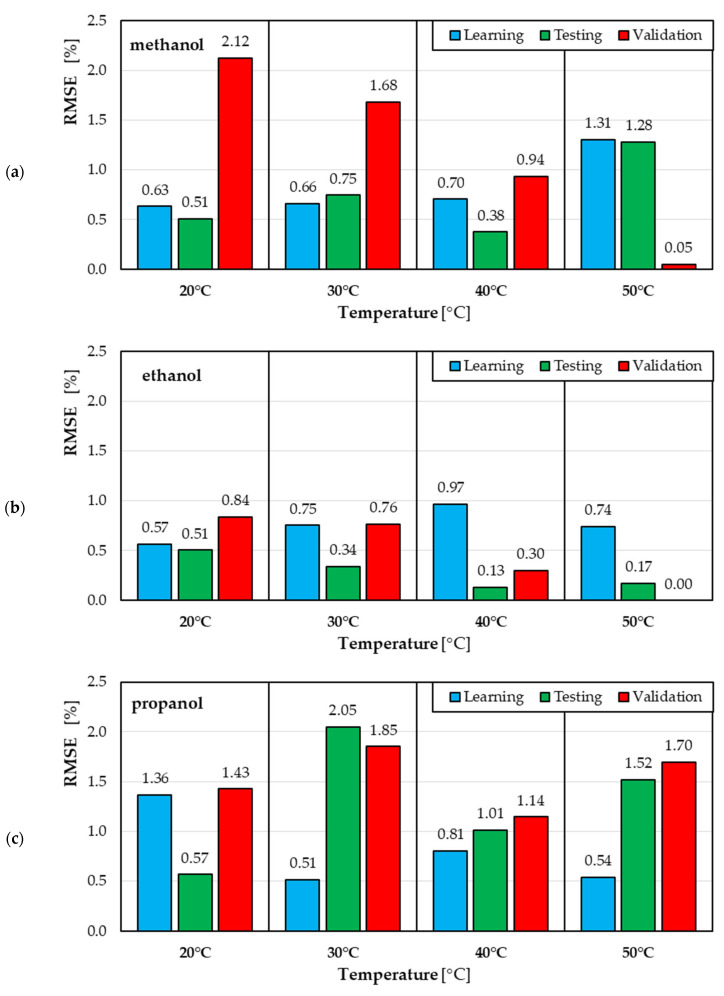
RMSE values calculated for individual solvents and different synthesis temperatures: (**a**) methanol, (**b**) ethanol and (**c**) propanol.

Figure 7 shows the RMSE values for individual samples (three different solvents, four synthesis temperatures) for the Training, Testing, and Validation subsets. These values were calculated for the selected ann08 neural network.

For the selected ann08 (MLP 5-8-1) neural network model, the sensitivity analysis results (error ratio values) are as follows (for the training, test, and validation subsets):

- for the particle size variable, the error ratio is 83.7;
- for the temperature variable, the error ratio is 56.1;
- for the solvent variable, the error ratio is 16.1.

Therefore, the most significant input variable is particle size. Temperature is slightly less significant, and solvent is the least significant. Error ratio values significantly greater than 1 indicate that all input variables are important for the ANN’s accuracy.

## 4. Discussion

In this work, RSME was adopted as a parameter defining the quality of neural models. The value of this error for the test subset was the criterion for selecting the best ANN. The lowest RMSE value of 0.98% was achieved by the ann08 neural network (Figure 2). This is an MLP network with five input neurons, eight neurons in the hidden layer, and one output neuron. For the remaining neural models, the error values did not differ significantly for either the training or test subsets. For the training subset, the RMSE varied from 0.84% to 2.26%, while for the test subset, the RMSE varied from 0.98% to 2.53%.

The RMSE values for the validation subset, which was not used in creating the neural models, are similar and vary from 1.27% to 2.36% (Figure 3). For the selected ann08 model, the error value is also the lowest (RMSE = 1.27%).

Comparison of the ZnO nanoparticle size distribution for different synthesis temperatures and solvents (Figure 4, Figure 5 and Figure 6) shows that the ANN model satisfactorily describes this process. For methanol (Figure 4), the best fit was obtained for a temperature of 20°C, and the worst fit was obtained for a temperature of 30°C (points from the validation subset ranged from 700 nm to 1000 nm). For ethanol (Figure 5), the worst fit was obtained for a temperature of 40 °C (particle size approximately 350 nm). For the remaining synthesis temperatures, the model fit to the experimental data was good. The worst fit of the ANN model to the measurement results was observed for propanol (Figure 6). Clear discrepancies were observed for all temperatures. For temperatures of 20 °C, 30 °C and 40 °C, these discrepancies occur for particle sizes ranging from 200 nm to 400 nm. For temperatures of 50 °C, the largest errors in model fit to the data occur for particle sizes ranging from 400 nm to 800 nm.

A more detailed analysis of the goodness-of-fit of the ann08 neural model to the experimental data can be performed by analyzing the RMSE values for individual solvents, synthesis temperatures, and the subsets to which individual measurement results were assigned (Figure 7).

The smallest error values occur for ethanol (Figure 7b). For the training subset, the RMSE varies from 0.57% at 20 °C to 0.97% at 40 °C. For the testing subset, the minimum RMSE is 0.13% at 40 °C, and the maximum is 0.51% at 20 °C. For the validation subset, the RMSE varies from 0.30% at 40°C to 0.84% at 20 °C. At 50 °C, RMSE = 0 indicates no calculated error (there was no experimental data in the validation subset).

The highest error values occur for propanol (Figure 7c), confirming that for this solvent, the neural model describes the synthesis process the worst. For the training subset, the RMSE varies from 0.51% at 30 °C to 1.36% at 20 °C. For the test subset, the minimum RMSE is 0.57% at 20 °C, and the maximum is 2.05% at 30 °C. For the validation subset, the RMSE varies from 1.14% at 40°C to 1.85% at 30 °C.

For ethanol, the range of RMSE values is intermediate, with the exception of the error at 20 °C in the validation subset (Figure 7a). For the training subset, the RMSE varies from 0.63% at 20 °C to 1.31% at 50 °C. For the test subset, the minimum RMSE is 0.38% at 40C and the maximum is 1.28% at 50C. For the validation subset, the RMSE varies from 0.05% at 50C to 2.12% at 20C.

Only a few publications describing research using ANNs to model the nanoparticle synthesis process have been found in the literature. However, the ANNs developed here model this process differently. Therefore, it is difficult to compare the results obtained by other authors.

Iliaz and co-authors [66] used an ANN to model the synthesis of optimized doxorubicin-loaded zinc oxide nanoflowers (DOX-ZnO-NF). They used NaOH Molarity, Sonication Temperature, and SonicationTime as ANN input variables. They adopted particle size, polydispersity index (PDI), zeta potential, yield, and encapsulation efficiency as the ANN output variables. The feed-forward neural network they developed had three neurons in the input layer, six neurons in the hidden layer, and five neurons in the output layer. They used the hyperbolic tangent as the activation function. Unfortunately, they only provide the sum of absolute error values, which for different data files range from 4.95 to 5.61.

Vaferi et al. used MLP-type ANN to predict the size of agglomerations in water–clay nanofluids [62]. The authors used 345 data points from 29 literature sources to create neural models. These data were divided into a subset used for ANN training (283 cases) and a subset used for testing the developed neural model (62 cases). In the ANN model, the following input variables were assumed: alumina dose (vol.%), surfactant concentration (wt.%), pH (-), ultrasonic time (min), ultrasonic power (W), ultrasonic frequency (kHz) and temperature (K). The output variable of the ANN was agglomeration size (nm). The best MLP model had nine neurons in the hidden layer, a tangent sigmoid activation function in the hidden layer, and a logarithm sigmoid activation function in the output layer. For this ANN, the RMSE was 646.23 (for the learning subset) and 300.35 (for the testing subset).

Khataman et al. developed an ANN model to predict the performance of the catalytic degradation process of 4-nitrophenol using ZnO nanoparticles supported on zeolites [57]. They used pH, initial concentration of 4-nitrophenol degradation, and exposure time as input variables for the ANN. They adopted 4-nitrophenol degradation in % (total organic compound) as the output variable. The model they developed is a three-layer MLP ANN with three neurons in the input layer, four neurons in the hidden layer, and one neuron in the output layer. Unfortunately, the authors only report an R^2^ value of 0.982 for the test set.

Melaibari et al. used ANN to predict the rheological behavior of hybrid antifreeze containing graphene oxide and copper oxide nanomaterials [58]. The authors used temperature, shear rate, and mass fraction as inputs to the ANN. They assumed viscosity (cP) as the network output. The value of the mean square error for the developed ANN was 0.0125 (corresponding to RSME = 0.1118).

Muneer et al. used ANN to predict the zeta potential, which is crucial in assessing the stability of nanofluids [68]. The literature data (249 cases) were divided into learning and testing subsets in the proportions of 80% and 20%. As the ANN input variables they used the following: silica nanoparticle size (nm), nanoparticle concentration (wt%), temperature (°C), salinity (ppm), pH, and media (qualitative variable: water, sand, glass, beads, coal, kaolinite). The output variable of the ANN was the Zeta potential (mV). The feed-forward ANN they developed had eleven neurons in the input layer, ten neurons in each of the two hidden layers, and one neuron in the output layer. The hidden layers used the hyperbolic tangent as the activation function, and the output layer used a pure linear activation function. The RMSE values calculated for the developed ANN model were 2.754 (for learning data) and 5.347 (for test data).

Researchers from Guizhou Normal University, Guiyang, and Peking University, Beijing (Jiwei Hu, Yu Hou, Jimei Qi, Xionghui Wei, and others), published two papers on the use of ANN to model the process of dye elimination (ethyl violet and crystal violet) from wastewater.

The first article published in the *Materials* journal reports on research in which an ANN was developed to model the crystal violet (CV) removal process using bimetallic Fe/Ni nanoparticles supported by reduced graphene oxide [56]. The ANN input variables used were initial dye concentration, initial pH, contact time and temperature, and the CV removal efficiency was used as the output variable. The authors developed a three-layer MLP ANN with four neurons in the input layer, three neurons in the hidden layer, and one neuron in the output layer. The experimental data (29 cases) were divided into a training subset (24 cases) and a testing subset (5 cases). For the developed ANN model, the RMSE values were 0.222% (for the learning subset) and 0.442% for the testing subset.

The second article published in the journal *Processes* contains the results of research in which the authors developed a back propagation ANN to model the process of ethyl violet elimination in wastewater using mesoporous, iron nanoparticle-doped, modified, and reduced graphene oxide [55]. They used initial EV concentration, initial pH, sonication time (contact time) and dosage as input variables for the ANN. The output variable of the ANN was the removal efficiency (%). The developed ANN had four input neurons, five hidden layer neurons, and one output layer neuron. For the training data, the mean square error was 0.00307 (RMSE = 0.055).

The above analysis of ANN applications in modeling nanoparticle-related problems shows that the errors associated with prediction results using neural models vary significantly. This depends on the problem being modeled and the data used to create the ANN model.

In the presented method of electrochemical synthesis of zinc oxide nanoparticles, the type of alcoholic solvent plays a key role in determining the form of the final product. The results of zinc oxide nanoparticle analysis [27] show that the largest particles, but also those with the greatest size variation, are formed in a methanol solution at 20 °C. Nanoparticles formed in an ethanol environment are the least diverse in terms of size and are also significantly smaller than those formed in methanol. In a propanol solvent at 20 °C, the zinc oxide nanoparticles obtained are the smallest and again show a wide range of sizes.

There is therefore a clear trend that the bigger the number of carbon atoms in an alcohol molecule, the smaller the zinc oxide nanoparticles formed in the alcohol environment. Larger alcohol molecules exhibit stronger intermolecular interactions (van der Waals interactions, hydrogen bonds). Stronger intermolecular interactions in a liquid directly translate into the viscosity of that liquid, which is the result of friction between molecules. According to the substance data sheets, the dynamic viscosity of methanol at 20 °C is η = 0.544-0.59 mPa∙s, ethanol η = 1.2 mPa∙s, and 1-propanol η = 2.3 mPa∙s. The addition of 5% water by volume to each of the alcohols will change the dynamic viscosity value to a small extent, increasing it in the case of methanol and decreasing it in the case of the other two alcohols.

In the course of electrode reactions and reactions occurring in the electrode space, as well as subsequent reactions taking place in the electrolyte volume, the transport of charges carried by ions plays a very important role. Due to the low dynamic viscosity of the methanol solution, charge-carrying ions can move much more freely in the solution volume than in ethanol and propanol solutions, which have higher viscosity. The electrochemical synthesis of zinc oxide nanoparticles in electrolytes with alcoholic solvents involves more than just detaching zinc cations from the electrode surface. The formation of zinc oxide nanoparticles is the result of a series of successive reactions taking place in the solution volume. The low resistance to the movement of the ions involved in these reactions results in greater efficiency. This allows the formation of large nanoparticles, but at the same time, due to the dynamics of the process, particles with a wide range of sizes are created.

The higher viscosity of the media in which the charge carriers involved in the reactions move, i.e., in ethanol and propanol synthesis environments, creates greater resistance to movement. This makes the process less dynamic in each subsequent alcoholic solvent. This is confirmed by observations and measurements of the quantities of zinc oxide nanoparticles formed as a result of synthesis. The impeded movement of ions involved in the reactions not only results in a decrease in the efficiency of the process. The resulting nanoparticles are also less diverse in size, especially in ethanol solution.

For most liquids (including water and alcohols), an increase in temperature causes a decrease in their dynamic viscosity. Supplying heat to a volume of liquid leads to an increase in the speed of movement of the liquid molecules and a decrease in their mutual interaction. This also increases the mobility of ions in the electrolyte involved in the formation of zinc oxide nanoparticles. Increasing the synthesis temperature caused a change in the size distribution of nanoparticles in each case. One can only speculate as to why increasing the process temperature promotes the formation of nanoparticles with greater size uniformity. To explain this phenomenon completely, it would be necessary to conduct research on the kinetics of reactions occurring under specific conditions.

The developed neural model (ann08) can be used to simulate the electrochemical synthesis of ZnO nanoparticles at various temperatures. It should be noted that the model is valid for temperatures ranging from 20 °C to 50 °C and for the solvents considered in its development (methanol, ethanol, and propanol).

## 5. Conclusions

1. The developed neural model, which allows for the prediction of the distribution of ZnO nanoparticles obtained in the electrochemical synthesis process, is an MLP neural network. During the formulation of the semantic model, it was assumed that the input (independent) variables were particle size (nm), solvent type, and temperature (°C), and the output (dependent) variable was fraction share (%).

2. The best neural network, ann08, for three input variables has five neurons in the input layer, eight neurons in the hidden layer, and one neuron in the output layer. This network has a hyperbolic tangent activation function for the neurons in the hidden layer and an exponential activation function for the neuron in the output layer.

3. SNN ann08 has a root mean square error 0.84% for the training subset, 0.98% for the testing subset, and 1.27% for the validation subset. The RMSE values are therefore small, which enables practical use of the ANN model.

4. The results of the sensitivity analysis confirmed that all input variables were significant for the accuracy of the developed ANN.

5. Although ANNs are used in nanoparticle research, no studies similar to ours have been found in publications on the use of ANNs to model various processes related to the production and use of nanoparticles. Our methodological approach to developing an ANN and modeling the ZnO synthesis process (formulating a semantic model, selecting the ANN type and activation function, conducting the training process, and selecting the ANN, and validating the model) is original and novel.

## Figures and Tables

**Table 1:** Activation functions of ANN neurons [39].

Function Name	Function Pattern	*y* Range
logistic	$y=\frac{1}{1+e^{-a}}$	$\left(0,+1\right)$
hyperbolic tangent	$y=\frac{e^{a}-e^{-a}}{e^{a}+e^{-a}}$	$\left(-1,+1\right)$
exponential	$y=e^{-a}$	$\left(0,+\infty \right)$

***a*** is the net input of a neuron (for MLPs—weighted sum of neuron’s inputs).

## Data Availability

The original contributions presented in this study are included in the article. Further inquiries can be directed to the corresponding authors.
